# Pasteurization of Milk

**Published:** 1913-10

**Authors:** Geo. Lloyd Magruder


### Pasteurization of Milk.

Pasteurization is a term applied to the process of heating milk to an appropriate temperature and holding it at that temperature for a sufficient time to destroy the disease-producing germs found in milk. Ayres has shown that all of the lactic-acid germs, which cause the souring of milk, are not destroyed. Pasteurization should not be confused with sterilization. Sterilization means the destruction of all germs, and requires heating to the boiling point on three successive days. The term Pasteurization is derived from the experiments made by Pasteur for the purpose of preventing the souring of wine and beer. He found that temperatures ranging from 122 degrees to 140 degrees F. were sufficient to prevent abnormal fermentation in them. Then investigations of General Sternberg, confirmed repeatedly by investigators of the highest authority in Europe and America, have shown that disease-producing germs are made harmless by a temperature of 140 degrees maintained for 20 minutes or more. It has also been established that this degree of heat and time of exposure do not change the taste nor exert any appreciable deleterious effects upon the nutritive value of milk. This is conclusively shown by Kastle and Roberts, in Bulletin 56, Bureau of Public Health. The cream line is not destroyed. It requires a much higher temperature to prevent the rising of the cream to the surface, the visual test of the richness of the milk. The higher temperatures keep the cream mixed with the milk.

### METHODS OF PASTEURIZATION.

The two methods of pasteurization are the flash and the holding processes. In the flash process the milk is heated to the required temperature and held at it for 30 seconds to 1 minute. This is not at all satisfactory and should not be allowed. In the holding process the milk is heated to 140 degrees or higher, and held at that temperature for 20 minutes or longer. To insure reliable pasteurization, that is, complete destruction of disease-producing germs, progressive milk dealers hold the milk at 145 degrees F. for 30 minutes. By the holding process properly conducted it is usual to destroy 99.93 to 99.99 per cent of the bacteria. Bulletin 56, Bureau of Public Health, and the records of the Health Department of the District of Columbia, show that counts as low as 2,000 per cubic centimeter have been secured by this method. From the fact that all bacteria are not killed in the process of pasteurization, it is equally as imperative that pasteurized milk, too, should be promptly cooled and kept cold with the same care that raw milk should be treated to check the growth of germs.

The splendid results obtained at infant-milk depots in securing exceedingly low counts, where milk has been properly pasteurized in the feeding bottles, has drawn attention to the desirability of pasteurizing milk in the bottles in which the milk is to be delivered to the consumer. This method of pasteurizing in the final container has many points of merit. It pasteurizes the bottle at the same time that the milk is pasteurized; it eliminates the possibility of re-infection from the handlers of the milk and dust settling upon the surface of the caps.

Dr. Charles E. North has shown that this process is practical from a commercial standpoint. He experimented by placing the milk in bottles, capping them with metal caps similar to those used for beer bottles, immersing them in water heated to 148 degrees for 15 minutes, and then cooling them to below 50 degrees.

The results obtained were most gratifying. About three-fourths of the bottles used showed counts of germs not exceeding 500 to the cubic centimeter; some were as low as 100; several were free from germs; the cream line promptly appeared. Ayres, of the Dairy Division, Department of Agriculture, expresses approbation of this method of pasteurization and is making investigations as to the feasibility of bottling and capping milk whilst it is hot. This will enable those who have large investments in other machinery to avail themselves of the metal caps.—*Geo. Lloyd Magruder.*

